# The Role of Medicare Insurance Coverage Type in Annual Wellness Visits: A Comparison Between Traditional Medicare and Medicare Advantage Plan

**DOI:** 10.1007/s11606-025-09825-8

**Published:** 2025-10-07

**Authors:** Zhang Zhang, Nancy L. Schoenborn, Katherine E. M. Miller, Jennifer L. Wolff, Daniel Polsky

**Affiliations:** 1 Department of Health Policy and Management, Johns Hopkins Bloomberg School of Public Health, Baltimore, MD USA; 2 Division of Geriatric Medicine and Gerontology, Department of Medicine, Johns Hopkins University School of Medicine, Baltimore, MD USA; 3 Johns Hopkins Carey School of Business, Baltimore, MD USA; 4 VA Partnered Evidence-based Policy Resource Center, Boston VA Health Care System, Boston, MA USA

## Abstract

**Background:**

The Medicare Annual Wellness Visit (AWVs) was introduced in 2011 as a preventive services visit. AWV uptake has been increasing but remains disproportionately low among vulnerable populations in Traditional Medicare (TM). However, less is known about the differential uptake of the AWV by Medicare insurance coverage type—a consequence of the increasing beneficiary enrollment shifts from TM to Medicare Advantage (MA) plans.

**Objective:**

This study aims to quantify the differential effects of Medicare insurance coverage type (MA versus TM) on AWV uptake for key subpopulations.

**Design:**

We used 20% nationally representative Medicare insurance claims data from 2018 to 2019. Probit models assessed the likelihood of AWV uptake, with subgroup analyses by age, race/ethnicity, dual eligibility, chronic conditions, and ADRD status.

**Participants:**

We included 8,799,206 Medicare beneficiaries aged 65 and older, among whom 41.2% were enrolled in MA, and 58.8% were enrolled in TM.

**Main Measures:**

The outcome is whether to have an AWV; the independent variable is the Medicare insurance coverage type.

**Key Results:**

Over 1/3 (37.3%) of beneficiaries received an AWV in 2019. MA enrollees were 4.3 percentage points more likely to receive an AWV than TM enrollees (*p* < 0.001). Subgroup analysis showed higher AWV uptake in MA across all key subgroups of interest (all *p* < 0.001), with the largest differences among the oldest-old adults aged above 85 + (5.6 percentage points), dual eligibles (11.5 percentage points), Black beneficiaries (8.9 percentage points), and those with ADRD (6.6 percentage points).

**Conclusion:**

Enrollment in an MA plan is associated with a higher probability of AWV uptake, particularly among vulnerable populations from racial and ethnic minorities, dual eligibility, and those diagnosed with ADRD. These findings highlight MA’s potential role in promoting preventive care and health equity. Future studies need to examine whether higher AWV uptake leads to improved patient outcomes in MA plans.

**Supplementary Information:**

The online version contains supplementary material available at 10.1007/s11606-025-09825-8.

## INTRODUCTION

The rapid growth of beneficiary enrollment in Medicare Advantage (MA), a private health plan alternative to the Traditional Fee-For-Service (TM) Medicare plan, has reshaped preventive healthcare delivery.^3,4^ By 2024, approximately 54% of Medicare beneficiaries were enrolled in MA plans.^1,5^ Compared with the TM plan, MA plans are specifically designed to encourage preventive care and coordinated care for older adults through risk-adjusted capitated payments.

The Annual Wellness Visits (AWVs), introduced in 2011 as part of the Affordable Care Act,^6–8^ are a key channel for delivering preventive care under Medicare. The AWV aims to promote health maintenance and support healthy aging by creating personalized preventive care plans: beneficiaries incur no copayments or deductibles after their first year of enrollment.^9^ Additionally, AWVs require completing a health risk assessment, which helps identify health risks that can benefit from early detection and prevention.^3,10–14^ Prior studies indicate that AWVs are associated with increased utilization of preventive services, including vaccinations, cancer screenings, and depression assessments.^15^ AWVs have also been shown to improve documentation of chronic conditions, functional impairments, and cognitive status, which are factors crucial for care planning in older adults.^16,17^ Further, AWVs have been associated with earlier diagnosis of conditions such as mild cognitive impairment (MCI) or Alzheimer’s disease and related dementias (ADRD), as well as reduced hospitalizations and emergency department visits among high-risk patients.^17–19^ In addition to supporting early detection, AWVs may also contribute to improved patient-provider communication and higher satisfaction with care.^20,21^ Despite the potential benefits of AWVs, the uptake rate among TM beneficiaries remains suboptimal.^11,22–24^ Previous studies show that the uptake rate of AWVs was a modest 7% completion rate in 2011 and improved to 20% in 2014 in the Traditional Medicare.^25,26^ 6.2% of Medicare Advantage enrollees had an AWV in 2011, the first year the benefit was offered, and that share quadrupled to 25.2% in 2015.^27,28^ AWV uptake also varies widely across sub-population groups, with lower rates of AWVs among those with lower income, racial and ethnic minorities, and those with medical complexity, including cognitive impairment.^23,29–31^ Compared to TM, MA plans provide additional incentives toward AWV uptake. In theory, the capitated per-member-per-month payment to MA plans for their beneficiaries encourages MA plans to lower spending and increase revenue.^32,33^ They may lower costs by developing strategies that can improve health and reduce the total costs of their enrollees.^32,33^ Providers in MA plans may increase revenue via greater risk-adjusted capitated payments if the risks of the beneficiaries are more thoroughly captured.^32,33^ The AWV may be one of those strategies, as the focus on preventive care and health risk assessment via AWV may prevent higher downstream costs that might be incurred by the plan and may be an important opportunity to document health risks and diagnoses.^3,10,11^ AWV uptake is particularly important for marginalized populations, such as racial and ethnic minorities and dual eligibles in Medicaid, whose health risks are more likely to remain undetected due to suboptimal access to preventive care.^3,10,11,34^ Thus, the incentive structure of MA may lead to differences in the AWV uptake between MA and TM beneficiaries, which may also have equity implications.

Recent survey-based evidence indicates that MA enrollees were 20% more likely to report having an AWV and 8.6% more likely to have a structured cognitive assessment compared with TM enrollees.^23^ However, these findings rely on self-reported data and are prone to recall errors, social desirability bias, and unrepresentative selection in the survey. While low AWV uptake among subgroups with lower income, racial and ethnic minorities, and those with medical complexity is well-documented, limited research has examined the role of insurance coverage in AWV uptake across diverse populations. Studies indicate that Black/Hispanic and dual-eligible beneficiaries are more inclined toward enrolling in MA.^35^ However, little is known about whether MA is associated with higher AWV uptake than TM among these populations. Additionally, MA plans have the flexibility to offer supplemental benefits, such as transportation and food assistance, to address social determinants of health that are identified through the AWV and are more common in underserved groups.^36^

Therefore, to address these gaps, this study used a nationally representative sample of TM and MA insurance claims data to examine the relationship between Medicare insurance coverage type and AWV uptake. We also analyzed the differential effects across key subgroups, including dual eligibles vs non-dual eligibles, racial/ethnicity groups, age groups, ADRD vs. non-ADRD, and comorbidity vs. non-comorbidity. We hypothesized that beneficiaries in MA plans will experience higher AWV uptake than TM, and we explored the differential effect across sub-population groups. We expected that MA plan enrollment would contribute to addressing the disparity in AWV uptake among those more vulnerable population groups, including the dual eligibles, the Black/Hispanic, and ADRD.

## METHODS

### Sample and Data

**Figure 1:**
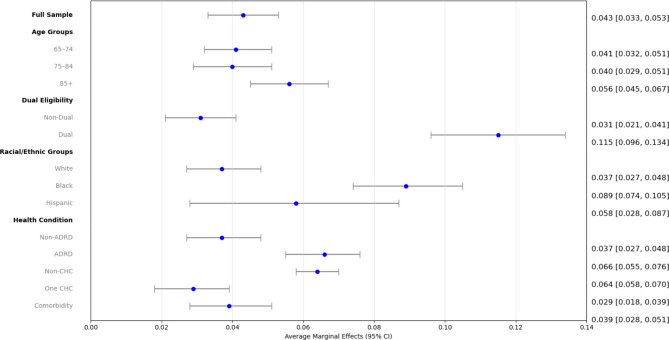
Difference in the likelihood of AWV uptake comparing MA versus TM plans and stratified by subgroups in 2019. This figure presents a comparison of the likelihood of AWV uptake between Medicare Insurance Coverage type (enrolled in MA or not) in 2019 and differential effects across sub-population groups. The blue dot represents the average marginal effects of the probit model with a cluster of each county, and the gray bar represents the 95% confidence interval. Please refer to the full results in the supplementary Table 1–6. Abbreviations: TM, Traditional Medicare Plans; MA, Medicare Advantage Plans; AWV, annual wellness visits; ADRD, Alzheimer’s disease and related dementias; CHC, chronic health conditions; Dual, dual eligibility.

The data were derived from similarly structured Medicare claims for both TM and MA beneficiaries. The encounter carrier file was used for 20% of MA beneficiaries, and 20% of TM beneficiaries. Our sample inclusion criteria applied to the Medicare Master Beneficiary Summary File included beneficiaries 65 years and older who were continuously enrolled in MA or TM without switching plans in 50 states or Washington, D.C., throughout 2019 with non-missing data on demographic and socioeconomic covariates. We selected 2019 as the year for which we have the most recent pre-COVID-19 data, avoiding potential confounding factors due to the pandemic. We used 2018 data to establish the comorbidity and ADRD status of the included beneficiaries. The sample selection table is in Appendix Table 2 and Fig. 1.

This study was deemed exempt by the Johns Hopkins Bloomberg School of Public Health Institutional Review Board.

### Measure

Our main outcome is receiving an Annual Wellness Visit (AWV) during the calendar year 2019. We defined dichotomous indicators of AWV use (yes/no) by Health Care Common Procedure Coding System (HCPCS) codes G0428 and G0439. HCPCS codes are comparably reported for MA and TM plans.^37,38^

Our key explanatory variable is Medicare insurance coverage type: TM or MA. Patient-level covariates include age, sex, dual eligibility status, Census region, and race and ethnicity, which were drawn from the Master Beneficiary Summary Files (MBSF). We used the MBSF’s Research Triangle Institute race variable to identify beneficiary race and ethnicity. We identified chronic conditions using Chronic Conditions Warehouse (CCW) algorithms. We included acute myocardial infarction, anemia, asthma, atrial fibrillation, cataracts, heart failure, chronic kidney disease, cancer, chronic obstructive pulmonary disease and Bronchiectasis, depression, diabetes, hypertension, hyperlipidemia, ischemic heart disease, osteoporosis, stroke, and ADRD.

### Analysis

We used probit models to analyze the relationship between Medicare insurance coverage type and receipt of AWV in 2019. We chose the probit model because it models the errors as close to a normal distribution, while the probit model can present the data better than the logistic model. We included clustering standard errors at the county level to adjust for within-county correlation in the outcome. We stratified sub-population groups by age (65–74, 75–84, and 85 +), dual eligibles (yes/no), and race/ethnicities (the White, the Black, the Hispanic, the Asian, the Native American, and others). We also examined the differential effects across sub-population groups by ADRD diagnosis (yes/no) and physical comorbidity status (none, one, or two or more). Interaction terms were not tested before stratification; rather, stratified models were based on hypotheses and used to explore variation in associations within each subgroup of interest. Our results are presented as the average marginal effects (AME) derived from the fitted regression models. AMEs were calculated using the delta method to estimate the change in the predicted probability of the outcome associated with a one-unit change in the covariate (or for a discrete change in the binary variables), holding other covariates at their observed values.

We adopted the Ordinary Least Squares linear regression model for sensitivity analysis. We also estimated multiple extensions to assess the robustness of estimates to different model specifications, including adding demographic factors (age, sex, and race/ethnicity), socioeconomic factors (census region and dual eligibility status), and health conditions (physical comorbidity and ADRD).

## STUDY RESULTS

### Descriptive Analysis

Table 1 presents the descriptive statistics of the 8,799,206 beneficiaries included in the analytic sample. Our sample consisted of 5,178,277 (58.8%) TM beneficiaries and 3,620,929 (41.2%) MA beneficiaries. Overall, 37.3% of beneficiaries received an AWV in 2019, with a higher AWV uptake among MA than TM beneficiaries (39.5% vs. 35.7%).

**Table 1:** Summary Statistics: Characteristics of the Sample

	**Total**	**TM**	**MA**	***p* value**
	*N* = 8,799,206 (100%)	*N* = 5,178,277	*N* = 3,620,929	
**Outcome variable**				
**AWV**				< 0.001
No	5,520,008 (62.7%)	3,329,367 (64.3%)	2,190,641 (60.5%)	
Yes	3,279,198 (37.3%)	1,848,910 (35.7%)	430,288 (39.5%)	
**Demographic and social factors**				
**Sex**				
Female	4,962,878 (56.4%)	2,894,836 (55.9%)	2,068,042 (57.1%)	
Male	3,836,328 (43.6%)	2,283,441 (44.1%)	1,552,887 (42.9%)	
**Age group**				< 0.001
65–74	4,735,017 (53.8%)	2,782,940 (53.7%)	1,952,077 (53.9%)	
75–84	2,915,397 (33.1%)	1,683,272 (32.5%)	1,232,125 (34.0%)	
85 +	1,148,792 (13.1%)	712,065 (13.8%)	436,727 (12.1%)	
**Race**				< 0.001
NH White	6,816,391 (77.5%)	4,246,511 (82.0%)	2,569,880 (71.0%)	
NH Black	765,719 (8.7%)	360,654 (7.0%)	405,065 (11.2%)	
Hispanic	657,051 (7.5%)	258,409 (5.0%)	398,642 (11.0%)	
Asian	298,867 (3.4%)	145,403 (2.8%)	153,464 (4.2%)	
Native American	31,253 (0.4%)	24,633 (0.5%)	6,620 (0.2%)	
Others	229,925 (2.6%)	142,667 (2.8%)	87,258 (2.4%)	
**Region**				< 0.001
Northeast	1,582,570 (18.0%)	916,571 (17.7%)	665,999 (18.4%)	
Midwest	1,938,737 (22.0%)	1,170,086 (22.6%)	768,651 (21.2%)	
South	3,330,599 (37.9%)	2,043,074 (39.5%)	1,287,525 (35.6%)	
West	1,947,300 (22.1%)	1,048,546 (20.2%)	898,754 (24.8%)	
**Dual eligible**				< 0.001
No	7,615,030 (86.5%)	4,572,560 (88.3%)	3,042,470 (84.0%)	
Yes	1,184,176 (13.5%)	605,717 (11.7%)	578,459 (16.0%)	
**Health conditions**				
**ADRD**				< 0.001
No	7,557,401 (93.6%)	4,440,390 (93.3%)	3,117,011 (94.1%)	
Yes	514,135 (6.4%)	320,140 (6.7%)	193,995 (5.9%)	
**Comorbidity**				< 0.001
Non-chronic condition	1,156,693 (13.1%)	726,329 (14.0%)	430,364 (11.9%)	
One chronic condition	965,938 (11.0%)	562,785 (10.9%)	403,153 (11.1%)	
Comorbidity (two or more chronic conditions)	6,676,575 (75.9%)	3,889,163 (75.1%)	2,787,412 (77.0%)	

*TM* Traditional Medicare Plans, *MA* Medicare Advantage Plans

Compared to TM beneficiaries, MA beneficiaries had higher proportion of females (55.9% in MA vs. 55.9% in TM), Black (11.2% in MA vs. 7.0% in TM), and Hispanic individuals (11.0% in MA vs. 5.0% in TM); dual eligibles for Medicare and Medicaid (16.0% in MA vs. 11.7% in TM); and comorbidity status (77.0% in MA vs. 75.1% in TM). Beneficiaries with an incident ADRD diagnosis represent 6.7% of the TM beneficiaries and 5.9% of the MA beneficiaries. The TM and MA groups had relatively similar distributions by age group and census region.

### Regression Analysis

MA beneficiaries had a 4.3 percentage point higher probability of receiving an AWV compared to TM beneficiaries (i.e., as 35.7% of the mean among TM enrollees) (*p* < 0.001, 95% CI 0.033,0.053) (Appendix Table 4) in regression analyses that adjusted for age, sex, race, regions, dual eligibles, and comorbidity status. MA was associated with greater AWV uptake for all sub-populations examined, but the magnitude of the difference varied (Fig. 1). Across different age groups, MA was associated with an increased likelihood of AWV uptake with the highest magnitude for adults aged 85 + (5.6 percentage points, *p* < 0.001, 95% CI 0.045–0.067), followed by those aged 65–74 (4.0 percentage points, *p* < 0.001, 95% CI 0.029–0.051) and 75–84 (4.1 percentage points, *p* < 0.001, 95% CI 0.032–0.051) (Appendix Table 5).

MA was associated with a significantly higher probability of AWV uptake among dual eligibles (11.5 percentage points, *p* < 0.001, 95% CI: 0.096–0.134) than non-dual eligibles (3.1 percentage points, *p* < 0.001, 95% CI 0.021–0.041) (Appendix Table 6). Among racial groups, MA beneficiaries experienced higher likelihood of AWV uptake by 8.9 percentage points for Black individuals (*p* < 0.001, 95% CI 0.074–0.105), 5.8 percentage points for Hispanic individuals (*p* < 0.001, 95% CI 0.028–0.087), and 3.7 percentage points for White individuals (*p* < 0.001, 95% CI 0.027–0.048) (Appendix Table 7).

For individuals with diagnosed ADRD, MA was associated with a 6.6 percentage point higher likelihood of AWV uptake (*p* < 0.001, 95% CI 0.055–0.076), while among non-ADRD individuals, the increase was 3.7 percentage points (*p* < 0.001, 95% CI 0.027–0.048) (Appendix Table 8). Among individuals with chronic conditions, MA beneficiaries with comorbidity had a 3.9 percentage point higher likelihood of AWV uptake (*p* < 0.001, 95% CI 0.028–0.051), while those with one chronic condition had a 2.9 percentage point increase (*p* < 0.001, 95% CI 0.018–0.039). In comparison, individuals without chronic conditions had a 6.4 percentage point higher likelihood of AWV uptake (*p* < 0.001, 95% CI 0.058–0.070) (Appendix Table 9).

We performed several sensitivity checks, including using an Ordinary Least Squares linear regression model (Appendix Table 10). We also estimated multiple extensions to assess the robustness of estimates to different model specifications. To control the confounders, we added a multiple set of covariates by adding demographic factors (age, sex, and race/ethnicity), socioeconomic factors (census region and dual eligibility status), and health conditions (comorbidity) (Appendix Table 11). Results appear highly stable as our main findings.

## DISCUSSION

Our analysis shows that AWV uptake is 4.3 percentage points higher for Medicare beneficiaries enrolled in MA relative to TM. If extrapolated to the 80 million in Medicare, this suggests nearly 350,000 additional AWV visits. This is the first study to examine the relationship between insurance coverage type (MA vs. TM) and AWV uptake across key subgroups using Medicare insurance claim data. Consistent with previous literature, AWV uptake was higher among beneficiaries enrolled in MA plans. Given the large scale of the Medicare program, the differential uptake indicates a public health impact, which may have important implications for promoting equitable delivery of preventive care via AWVs. However, we also found that the effects varied across sub-population groups, and the magnitude was most pronounced in the oldest old, racial/ethnic minorities, dual eligibles, and those diagnosed with ADRD. Several features of MA plans provide mechanisms that may explain our findings.

On the positive side for public health, MA plans are incentivized to provide quality care, which might increase the use of AWVs. For one, they are incentives to prioritize preventive care through mechanisms like the Medicare Star Rating system, while there are no similar incentives in TM. This system ties plan performance to financial rewards and market competitiveness, with higher ratings resulting in bonus payments and greater plan attractiveness.^39^ AWVs, as a component of preventive care in evaluation metrics, are heavily emphasized within MA and drive efforts to prioritize completion to achieve better ratings and improve care quality metrics.^39^ Secondly, MA plans are paid via a capitated per-member-per-month payment per enrollee, which provides flexibility in care modalities and incentives for cost savings that encourage integrated care, coordinating services across primary care, specialists, and preventive care.^32,33^ This approach can reduce care fragmentation and ensure AWVs are incorporated into routine medical visits, particularly for individuals with chronic conditions or other complex needs.

On the potentially negative side for public health, the per-member-per-month risk-adjusted capitated amount per beneficiary, regardless of their service use, also encourages MA plans to ensure the risk of the beneficiaries is fully captured. The risk adjustment ensures that MA plans receive adequate funding to provide high-quality, affordable care to beneficiaries, particularly those with complex health needs. However, MA’s reliance on risk-adjusted payments introduces strong financial incentives to document diagnoses during AWVs, potentially inflating risk scores to secure higher reimbursements (upcoding problems).^40,41^ Upcoding occurs when providers inflate diagnoses to secure higher risk-adjusted payments, which could lead to financial inefficiencies and overpayments.^42^ The incentive for MA plans to identify and code risks for risk adjustment may also explain greater AWV uptake in MA plans.

While our analysis describes patterns of AWV uptake, it does not provide evidence regarding the clinical downstream outcomes of these visits, which will be explored in future work. It also does not assess whether AWVs in MA are associated with changes in diagnostic coding or risk scores. Future studies using longitudinal data and exogenous policy changes in risk adjustment in MA plans are needed to explore this issue as well.

Our findings reveal that MA is associated with greater increases in AWV uptake among vulnerable subgroups that have historically experienced lower AWV utilization, including dual-eligible beneficiaries, the Black and Hispanic, the oldest-old (≥ 85 years), and individuals with ADRD. These results underscore significant disparities in the AWV uptake between MA and TM beneficiaries, with MA consistently demonstrating higher AWV utilization rates across demographic and clinical subpopulations (Appendix Table 3). MA may not only be more effective in promoting preventive care but also potentially address disparities in preventive care utilization, particularly among populations with complex healthcare needs. MA’s differential effects among subgroups may have several possible explanations. MA plans frequently offer benefits beyond those covered by TM, such as transportation to medical appointments, home health visits, and culturally tailored care. These benefits can reduce barriers to accessing AWVs for low-income, disabled, or minority beneficiaries.^43,44^ This proactive approach can also ensure that beneficiaries with complex healthcare needs are more likely to participate in preventive services.

While study findings suggest MA successfully promotes AWV and reduces or even eliminates the lower AWV uptake among populations with higher clinical and socioeconomic complexity, such as dual eligibles, the Black and the Hispanic, and diagnosed ADRD, the alternative motivations for AWV raise questions as to the extent to which AWV translates into clinically meaningful benefit as opposed to strategic coding practices.^42^ While risk adjustment ensures appropriate funding for high-need beneficiaries, it also can contribute to overpayments driven by less clinically meaningful, albeit accurate, diagnoses. Therefore, further investigation is required to determine whether the higher uptake of AWVs in MA translates to improved patient outcomes.

## LIMITATIONS

We recognize several limitations of this study. First, there may be a concern that the MA encounter data is incomplete or contains errors, which have been subject to data quality issues.^45,46^ Second, our study is limited to the pre-COVID-19 pandemic period, which may not apply to the patterns during and after the pandemic. In our future work, we plan to use multiple years of data, including more recent years, to understand the relatively longer effect of insurance coverage types on AWV uptake. Third, we acknowledge that selection bias exists in MA and TM. Existing literature indicates that Black and dual-eligible beneficiaries are more inclined toward MA enrollment, potentially due to the additional benefits and care coordination that MA plans offer.^35^ We also acknowledge the omitted variable bias due to the use of administrative data. Key confounding variables, such as provider and clinical characteristics, or unmeasured health behaviors, may not be captured in claims-based datasets. This limitation potentially biases our estimates and the interpretation of the results. Fourth, our current study cannot fully understand if MA is associated with more new diagnoses of diseases or upcoding patients. In our future work, we will adopt a more rigorous investigation into changes in diagnoses or testing patterns during pre- and post-AWVs.

## CONCLUSION

In conclusion, MA plan enrollment was associated with higher AWV uptake, particularly among vulnerable populations from racial and ethnic minorities, dual eligibility, and those diagnosed with ADRD. These findings highlight MA’s potential role in promoting preventive care and health equity. Future studies need to examine whether higher AWV uptake leads to improved patient outcomes in MA plans and evaluate the downstream clinical benefits of AWV utilization, such as reductions in acute care utilization or improved chronic disease management, to determine whether AWVs lead to meaningful health outcomes. Balancing payment incentives with value-based care delivery remains critical to ensure that AWVs achieve their intended goals of equitable and efficient preventive care, particularly for the most vulnerable Medicare populations.

## Supplementary Information

Below is the link to the electronic supplementary material.

Supplementary Material 1 (DOCX 128 KB)

## Data Availability

Per the data use agreement, the data is not permitted to be shared.
