# Supplementary material for: The Role of Medicare Insurance Coverage Type in Annual Wellness Visits: A Comparison Between Traditional Medicare and Medicare Advantage Plan
Source: J Gen Intern Med. 2025 Oct 7;41(5):1276–82. doi: 10.1007/s11606-025-09825-8 (PMC13083681; doi:10.1007/s11606-025-09825-8)
Supplement: Supplementary file 1 — Supplementary Material 1 (DOCX 128 KB) [file 11606_2025_9825_MOESM1_ESM.docx]

**Appendix**

**Table 1. Sample selection**

|  | **2019** | |
| --- | --- | --- |
|  | **N** | **%** |
| **In the 20% Sample** | 12,886,340 | 100% |
| 65 or over | 11,100,468 | 86% |
| 50 states or Washington DC | 10,869,934 | 84% |
| Continuous enrollment in TM or MA for the 2019 calendar year | 9,259,954 | 72% |
| missing data in demographics and diseases | 8,799,206 | 68% |

**Figure 1. Sample Selection Flow Chart**


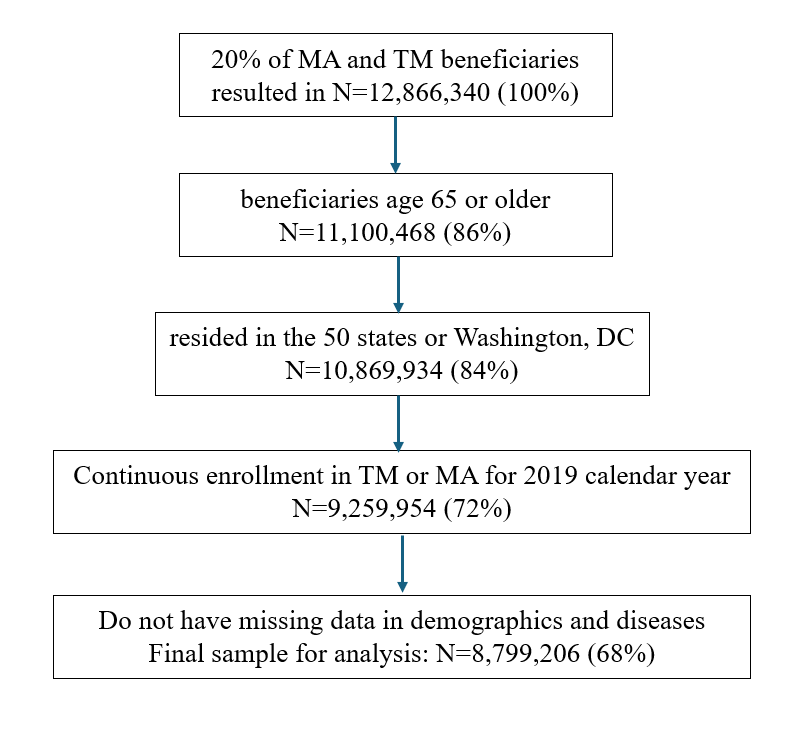


**Table 2. Summary: Annual Wellness Visit by Medicare Insurance Type across Sub-population group**

|  | **TM** | | | | | **MA** | | | | |
| --- | --- | --- | --- | --- | --- | --- | --- | --- | --- | --- |
| **Annual Wellness Visit** | **Total** | **No** |  | **Yes** |  | **Total** | **No** |  | **Yes** |  |
| (1=Yes; 0=No) | **N** | **N** | **%** | **N** | **%** | **N** | **N** | **%** | **N** | **%** |
|  | 5,178,277 | 3,329,367 | 64.29% | 1,848,910 | 35.71% | 3,620,929 | 2,190,641 | 60.50% | 1,430,288 | 39.50% |
| **Sex** |  |  |  |  |  |  |  |  |  |  |
| female | 2,894,836 | 1,816,087 | 62.74% | 1,078,749 | 37.26% | 2,068,042 | 1,232,658 | 59.61% | 835,384 | 40.39% |
| male | 2,283,441 | 1,513,280 | 66.27% | 770,161 | 33.73% | 1,552,887 | 957,983 | 61.69% | 594,904 | 38.31% |
| **Age group** |  |  |  |  |  |  |  |  |  |  |
| 65-74 | 2,782,940 | 1,808,801 | 65.00% | 974,139 | 35.00% | 1,952,077 | 1,194,061 | 61.17% | 758,016 | 38.83% |
| 75-84 | 1,683,272 | 1,039,824 | 61.77% | 643,448 | 38.23% | 1,232,125 | 723,325 | 58.71% | 508,800 | 41.29% |
| 85+ | 712,065 | 480,742 | 67.51% | 231,323 | 32.49% | 436,727 | 273,255 | 62.57% | 163,472 | 37.43% |
| **Race** |  |  |  |  |  |  |  |  |  |  |
| NH White | 4,246,511 | 2,677,241 | 63.05% | 1,569,270 | 36.95% | 2,569,880 | 1,529,847 | 59.53% | 1,040,033 | 40.47% |
| NH Black | 360,654 | 253,195 | 70.20% | 107,459 | 29.80% | 405,065 | 242,992 | 59.99% | 162,073 | 40.01% |
| Hispanic | 258,409 | 192,751 | 74.59% | 65,658 | 25.41% | 398,642 | 263,313 | 66.05% | 135,329 | 33.95% |
| Asian | 145,403 | 96,187 | 66.15% | 49,216 | 33.85% | 153,464 | 95,017 | 61.91% | 58,447 | 38.09% |
| Native American | 24,633 | 21,698 | 88.09% | 2,935 | 11.91% | 6,620 | 4,710 | 71.15% | 1,910 | 28.85% |
| others | 142,667 | 88,295 | 61.89% | 54,372 | 38.11% | 87,258 | 54,762 | 62.76% | 32,496 | 37.24% |
| **Region** |  |  |  |  |  |  |  |  |  |  |
| northeast | 916,571 | 562,303 | 61.35% | 354,268 | 38.65% | 665,999 | 407,036 | 61.12% | 258,963 | 38.88% |
| midwest | 1,170,086 | 755,923 | 64.60% | 414,163 | 35.40% | 768,651 | 447,638 | 58.24% | 321,013 | 41.76% |
| south | 2,043,074 | 1,272,181 | 62.27% | 770,893 | 37.73% | 1,287,525 | 718,193 | 55.78% | 569,332 | 44.22% |
| west | 1,048,546 | 738,960 | 70.47% | 309,586 | 29.53% | 898,754 | 617,774 | 68.74% | 280,980 | 31.26% |
| **Dual eligible** |  |  |  |  |  |  |  |  |  |  |
| No | 4,572,560 | 2,876,286 | 62.90% | 1,696,274 | 37.10% | 3,042,470 | 1,835,672 | 60.33% | 1,206,798 | 39.67% |
| Yes | 605,717 | 453,081 | 74.80% | 152,636 | 25.20% | 578,459 | 354,969 | 61.36% | 223,490 | 38.64% |
| **ADRD** |  |  |  |  |  |  |  |  |  |  |
| No | 4,440,390 | 2,781,066 | 62.63% | 1,659,324 | 37.37% | 3,117,011 | 1,855,235 | 59.52% | 1,261,776 | 40.48% |
| Yes | 320,140 | 231,735 | 72.39% | 88,405 | 27.61% | 193,995 | 127,450 | 65.70% | 66,545 | 34.30% |
| **Comorbidity** |  |  |  |  |  |  |  |  |  |  |
| Non-disease | 726,329 | 627,741 | 86.43% | 98,588 | 13.57% | 430,364 | 345,473 | 80.27% | 84,891 | 19.73% |
| One disease | 562,785 | 377,446 | 67.07% | 185,339 | 32.93% | 403,153 | 263,187 | 65.28% | 139,966 | 34.72% |
| Comorbidity | 3,889,163 | 2,324,180 | 59.76% | 1,564,983 | 40.24% | 2,787,412 | 1,581,981 | 56.75% | 1,205,431 | 43.25% |

**Abbreviations: TM - Traditional Medicare Plans; MA - Medicare Advantage Plans； ADRD - Alzheimer's Disease and Related Dementias； NH – Non-Hispanic**

**Table 3. AWVs Uptake and Medicare Insurance Type: Probit Model and Marginal Effects**

|  | **Coefficients** | **Average Marginal Effects (AME)** |
| --- | --- | --- |
|  | b/se/ci95 | ame/se/ci95 |
|  |  |  |
| Fee-for-Service | Ref | Ref |
| Medicare Advantage | 0.118^****^ | 0.043^****^ |
|  | 0.014 | 0.005 |
|  | 0.091,0.146 | 0.033,0.053 |
| 65-74 | Ref | Ref |
| 75-84 | -0.004* | -0.001* |
|  | 0.002 | 0.001 |
|  | -0.008,0.001 | -0.003,0.000 |
| 85+ | -0.125**** | -0.045**** |
|  | 0.004 | 0.001 |
|  | -0.134,-0.117 | -0.048,-0.042 |
| Female | Ref | Ref |
| Male | -0.075**** | -0.027**** |
|  | 0.002 | 0.001 |
|  | -0.079,-0.071 | -0.029,-0.026 |
| NH White | Ref | Ref |
| NH Black | -0.116**** | -0.042**** |
|  | 0.015 | 0.005 |
|  | -0.147,-0.086 | -0.052,-0.031 |
| Hispanic | -0.152**** | -0.054**** |
|  | 0.023 | 0.008 |
|  | -0.197,-0.108 | -0.070,-0.039 |
| Asian | 0.055 | 0.02 |
|  | 0.041 | 0.015 |
|  | -0.024,0.135 | -0.009,0.050 |
| Native American | -0.642**** | -0.202**** |
|  | 0.035 | 0.009 |
|  | -0.711,-0.572 | -0.220,-0.184 |
| Others | 0.042**** | 0.015**** |
|  | 0.011 | 0.004 |
|  | 0.021,0.063 | 0.008,0.023 |
| Northeast | Ref | Ref |
| Midwest | -0.013 | -0.005 |
|  | 0.032 | 0.012 |
|  | -0.076,0.050 | -0.028,0.018 |
| South | 0.046 | 0.017 |
|  | 0.03 | 0.011 |
|  | -0.012,0.103 | -0.004,0.038 |
| West | -0.191**** | -0.068**** |
|  | 0.045 | 0.016 |
|  | -0.279,-0.103 | -0.099,-0.037 |
| Non-Dual eligible | Ref | Ref |
| Dual eligible | -0.161**** | -0.057**** |
|  | 0.017 | 0.006 |
|  | -0.195,-0.127 | -0.069,-0.045 |
| Non-disease | Ref | Ref |
| One disease | 0.565**** | 0.172**** |
|  | 0.005 | 0.002 |
|  | 0.555,0.574 | 0.168,0.176 |
| Comorbidity | 0.779**** | 0.252**** |
|  | 0.008 | 0.003 |
|  | 0.762,0.795 | 0.245,0.258 |
| Non-ADRD | Ref | Ref |
| ADRD | -0.230**** | -0.081**** |
|  | 0.007 | 0.002 |
|  | -0.243,-0.217 | -0.086,-0.076 |
| Constant | -0.894**** |  |
|  | 0.025 |  |
|  | -0.942,-0.845 |  |
| Observations | 8799206 | 8799206 |

**Note:** *p < 0.10 **p < 0.05 ***p < 0.01 ****p < 0.001

**Abbreviations:** b: coefficient; se: standard error; ci95: 95% confidence interval; ame: Average Marginal Effects; NH – Non Hispanic

**Table 4. AWVs Uptake and Medicare Insurance Type by Age Group**

|  | **65-74** | **75-84** | **85+** |
| --- | --- | --- | --- |
|  | ame/se/ci95 | ame/se/ci95 | ame/se/ci95 |
| Fee-for-Service | Ref | Ref | Ref |
| Medicare Advantage | 0.041**** | 0.040**** | 0.056**** |
|  | 0.005 | 0.006 | 0.006 |
|  | 0.032,0.051 | 0.029,0.051 | 0.045,0.067 |
| Female | Ref | Ref | Ref |
| Male | -0.035**** | -0.022**** | -0.007**** |
|  | 0.001 | 0.001 | 0.001 |
|  | -0.037,-0.034 | -0.024,-0.020 | -0.010,-0.005 |
| NH White | Ref | Ref | Ref |
| NH Black | -0.045**** | -0.045**** | -0.017*** |
|  | 0.005 | 0.007 | 0.006 |
|  | -0.055,-0.035 | -0.057,-0.032 | -0.029,-0.004 |
| Hispanic | -0.056**** | -0.063**** | -0.023*** |
|  | 0.007 | 0.009 | 0.008 |
|  | -0.071,-0.042 | -0.081,-0.046 | -0.039,-0.006 |
| Asian | 0.018 | 0.013 | 0.047** |
|  | 0.014 | 0.015 | 0.021 |
|  | -0.010,0.046 | -0.016,0.042 | 0.007,0.087 |
| Native American | -0.206**** | -0.211**** | -0.157**** |
|  | 0.009 | 0.011 | 0.013 |
|  | -0.224,-0.189 | -0.231,-0.190 | -0.182,-0.132 |
| others | 0.025**** | -0.023*** | -0.013 |
|  | 0.003 | 0.008 | 0.01 |
|  | 0.018,0.031 | -0.038,-0.008 | -0.033,0.008 |
| Northeast | Ref | Ref | Ref |
| Midwest | -0.007 | -0.003 | -0.002 |
|  | 0.011 | 0.013 | 0.011 |
|  | -0.029,0.016 | -0.029,0.022 | -0.024,0.021 |
| South | 0.007 | 0.025** | 0.035**** |
|  | 0.01 | 0.012 | 0.011 |
|  | -0.013,0.027 | 0.001,0.049 | 0.014,0.056 |
| West | -0.071**** | -0.067**** | -0.060**** |
|  | 0.015 | 0.018 | 0.016 |
|  | -0.100,-0.043 | -0.102,-0.032 | -0.092,-0.029 |
| Non-Dual eligible | Ref | Ref | Ref |
| Dual eligible | -0.054**** | -0.056**** | -0.073**** |
|  | 0.005 | 0.007 | 0.008 |
|  | -0.064,-0.045 | -0.071,-0.042 | -0.088,-0.058 |
| Non-disease | Ref | Ref | Ref |
| One disease | 0.176**** | 0.172**** | 0.150**** |
|  | 0.002 | 0.002 | 0.003 |
|  | 0.172,0.180 | 0.167,0.176 | 0.145,0.156 |
| Comorbidity | 0.251**** | 0.260**** | 0.236**** |
|  | 0.003 | 0.004 | 0.004 |
|  | 0.245,0.257 | 0.253,0.268 | 0.228,0.244 |
| Non-ADRD | Ref | Ref | Ref |
| ADRD | -0.067**** | -0.077**** | -0.089**** |
|  | 0.003 | 0.003 | 0.002 |
|  | -0.072,-0.061 | -0.083,-0.072 | -0.093,-0.084 |
| Observations | 4735017 | 2915397 | 1148792 |

**Note:** *p < 0.10 **p < 0.05 ***p < 0.01 ****p < 0.001

**Abbreviations:** ame: Average Marginal Effects; se: Standard Error; ci95: 95% Confidence Interval

**Table 5. AWVs Uptake and Medicare Insurance Type by Dual Eligible**

|  | **Dual** | **Non-Dual** |
| --- | --- | --- |
|  | ame/se/ci95 | ame/se/ci95 |
| Fee-for-Service | Ref | Ref |
| Medicare Advantage | 0.115**** | 0.031**** |
|  | 0.01 | 0.005 |
|  | 0.096,0.134 | 0.021,0.041 |
| 65-74 | Ref | Ref |
| 75-84 | 0.004*** | -0.004**** |
|  | 0.002 | 0.001 |
|  | 0.001,0.007 | -0.006,-0.002 |
| 85+ | -0.033**** | -0.048**** |
|  | 0.002 | 0.002 |
|  | -0.037,-0.029 | -0.052,-0.045 |
| Female | Ref | Ref |
| Male | -0.026**** | -0.028**** |
|  | 0.002 | 0.001 |
|  | -0.030,-0.022 | -0.029,-0.026 |
| NH White | Ref | Ref |
| NH Black | 0.001 | -0.053**** |
|  | 0.006 | 0.005 |
|  | -0.010,0.013 | -0.063,-0.043 |
| Hispanic | -0.009 | -0.074**** |
|  | 0.012 | 0.007 |
|  | -0.033,0.014 | -0.088,-0.060 |
| Asian | 0.119**** | -0.021 |
|  | 0.015 | 0.014 |
|  | 0.089,0.148 | -0.049,0.006 |
| Native American | -0.154**** | -0.205**** |
|  | 0.013 | 0.009 |
|  | -0.179,-0.129 | -0.224,-0.187 |
| others | 0.044**** | 0.013*** |
|  | 0.013 | 0.004 |
|  | 0.019,0.068 | 0.004,0.021 |
| Northeast | Ref | Ref |
| Midwest | -0.002 | -0.006 |
|  | 0.011 | 0.013 |
|  | -0.023,0.019 | -0.031,0.019 |
| South | 0.051**** | 0.01 |
|  | 0.013 | 0.012 |
|  | 0.026,0.076 | -0.013,0.033 |
| West | -0.045** | -0.070**** |
|  | 0.02 | 0.017 |
|  | -0.084,-0.007 | -0.103,-0.037 |
| Non-disease | Ref | Ref |
| One disease | 0.145**** | 0.175**** |
|  | 0.003 | 0.002 |
|  | 0.138,0.151 | 0.172,0.179 |
| Comorbidity | 0.237**** | 0.254**** |
|  | 0.007 | 0.003 |
|  | 0.224,0.251 | 0.247,0.260 |
| Non-ADRD | Ref | Ref |
| ADRD | -0.092**** | -0.067**** |
|  | 0.005 | 0.002 |
|  | -0.102,-0.082 | -0.070,-0.063 |
| Observations | 1184176 | 7615030 |

**Note:** *p < 0.10 **p < 0.05 ***p < 0.01 ****p < 0.001

**Abbreviations:** ame: Average Marginal Effects; se: Standard Error; ci95: 95% Confidence Interval

**Table 6. AWVs Uptake and Medicare Insurance Type by Racial Group**

|  | **White** | **Black** | **Hispanic** |
| --- | --- | --- | --- |
|  | ame/se/ci95 | ame/se/ci95 | ame/se/ci95 |
| Fee-for-Service | Ref | Ref | Ref |
| Medicare Advantage | 0.037**** | 0.089**** | 0.058**** |
|  | 0.006 | 0.008 | 0.015 |
|  | 0.027,0.048 | 0.074,0.105 | 0.028,0.087 |
| 65-74 | Ref | Ref | Ref |
| 75-84 | -0.001 | 0.004** | -0.005** |
|  | 0.001 | 0.002 | 0.002 |
|  | -0.003,0.000 | 0.000,0.007 | -0.009,-0.001 |
| 85+ | -0.048**** | -0.024**** | -0.026**** |
|  | 0.002 | 0.003 | 0.003 |
|  | -0.051,-0.045 | -0.029,-0.019 | -0.031,-0.021 |
| Female | Ref | Ref | Ref |
| Male | -0.027**** | -0.044**** | -0.021**** |
|  | 0.001 | 0.002 | 0.002 |
|  | -0.028,-0.026 | -0.047,-0.041 | -0.025,-0.017 |
| Northeast | Ref | Ref | Ref |
| Midwest | -0.012 | 0.041* | 0.051** |
|  | 0.012 | 0.021 | 0.024 |
|  | -0.035,0.012 | -0.001,0.083 | 0.003,0.098 |
| South | 0.009 | 0.080**** | 0.034 |
|  | 0.011 | 0.019 | 0.029 |
|  | -0.013,0.031 | 0.043,0.117 | -0.023,0.091 |
| West | -0.068**** | -0.071*** | -0.042 |
|  | 0.016 | 0.025 | 0.026 |
|  | -0.100,-0.036 | -0.121,-0.022 | -0.094,0.009 |
| Non-Dual eligible | Ref | Ref | Ref |
| Dual eligible | -0.094**** | -0.031**** | -0.012 |
|  | 0.004 | 0.004 | 0.009 |
|  | -0.103,-0.086 | -0.039,-0.023 | -0.029,0.005 |
| Non-disease | Ref | Ref | Ref |
| One disease | 0.169**** | 0.176**** | 0.170**** |
|  | 0.002 | 0.004 | 0.005 |
|  | 0.165,0.173 | 0.169,0.183 | 0.161,0.179 |
| Comorbidity | 0.246**** | 0.286**** | 0.256**** |
|  | 0.003 | 0.005 | 0.008 |
|  | 0.239,0.252 | 0.276,0.295 | 0.240,0.273 |
| Non-ADRD | Ref | Ref | Ref |
| ADRD | -0.090**** | -0.055**** | -0.020**** |
|  | 0.002 | 0.004 | 0.004 |
|  | -0.094,-0.086 | -0.063,-0.048 | -0.029,-0.012 |
| Observations | 6816391 | 765719 | 657051 |

**Note:** *p < 0.10 **p < 0.05 ***p < 0.01 ****p < 0.001

**Abbreviations:** ame: Average Marginal Effects; se: Standard Error; ci95: 95% Confidence Interval

**Table 7. AWVs Uptake and Insurance Type by pre-diagnosed ADRD or not**

|  | **Non-ADRD** | **ADRD** |
| --- | --- | --- |
|  | ame/se/ci95 | ame/se/ci95 |
| Fee-for-Service | Ref | Ref |
| Medicare Advantage | 0.037**** | 0.066**** |
|  | 0.005 | 0.005 |
|  | 0.027,0.048 | 0.055,0.076 |
| 65-74 | Ref | Ref |
| 75-84 | -0.004**** | -0.011**** |
|  | 0.001 | 0.002 |
|  | -0.005,-0.002 | -0.015,-0.007 |
| 85+ | -0.045**** | -0.061**** |
|  | 0.002 | 0.003 |
|  | -0.048,-0.042 | -0.066,-0.055 |
| Female | Ref | Ref |
| Male | -0.029**** | 0.004*** |
|  | 0.001 | 0.001 |
|  | -0.031,-0.028 | 0.001,0.006 |
| NH White | 0.000 | 0.000 |
| NH Black | -0.049**** | 0.016*** |
|  | 0.006 | 0.006 |
|  | -0.060,-0.038 | 0.004,0.027 |
| Hispanic | -0.066**** | 0.037**** |
|  | 0.008 | 0.01 |
|  | -0.082,-0.050 | 0.017,0.057 |
| Asian | 0.011 | 0.114**** |
|  | 0.015 | 0.022 |
|  | -0.018,0.041 | 0.070,0.158 |
| Native American | -0.216**** | -0.103**** |
|  | 0.009 | 0.013 |
|  | -0.234,-0.197 | -0.128,-0.079 |
| others | 0.014**** | 0.036** |
|  | 0.004 | 0.016 |
|  | 0.006,0.022 | 0.005,0.068 |
| Northeast | Ref | Ref |
| Midwest | -0.004 | 0.003 |
|  | 0.013 | 0.008 |
|  | -0.029,0.020 | -0.014,0.019 |
| South | 0.016 | 0.044**** |
|  | 0.012 | 0.008 |
|  | -0.007,0.038 | 0.029,0.060 |
| West | -0.071**** | -0.038*** |
|  | 0.017 | 0.014 |
|  | -0.104,-0.039 | -0.065,-0.012 |
| Non-Dual eligible | Ref | Ref |
| Dual eligible | -0.056**** | -0.111**** |
|  | 0.006 | 0.008 |
|  | -0.067,-0.044 | -0.127,-0.096 |
| Non-disease | Ref | Ref |
| One disease | 0.180**** | 0.104**** |
|  | 0.002 | 0.003 |
|  | 0.176,0.184 | 0.098,0.110 |
| Comorbidity | 0.261**** | 0.177**** |
|  | 0.003 | 0.004 |
|  | 0.254,0.268 | 0.169,0.185 |
| Observations | 7557401 | 514135 |

**Note:** *p < 0.10 **p < 0.05 ***p < 0.01 ****p < 0.001

**Abbreviations:**ame: Average Marginal Effects; se: Standard Error; ci95: 95% Confidence Interval

**Table 8. AWVs Uptake and Insurance Type by Pre-diagnosed Comorbidity Status**

|  | **Non-Chronic Condition** | **One Chronic Condition** | **Comorbidity** |
| --- | --- | --- | --- |
|  | b/se/ci95 | b/se/ci95 | b/se/ci95 |
| Fee-for-Service | Ref | Ref | Ref |
| Medicare Advantage | 0.064**** | 0.029**** | 0.039**** |
|  | 0.003 | 0.005 | 0.006 |
|  | 0.058,0.070 | 0.018,0.039 | 0.028,0.051 |
| 65-74 | Ref | Ref | Ref |
| 75-84 | -0.006**** | -0.015**** | 0.001 |
|  | 0.001 | 0.001 | 0.001 |
|  | -0.008,-0.004 | -0.018,-0.013 | -0.001,0.003 |
| 85+ | -0.037**** | -0.062**** | -0.044**** |
|  | 0.001 | 0.002 | 0.002 |
|  | -0.040,-0.034 | -0.066,-0.058 | -0.047,-0.041 |
| Female | Ref | Ref | Ref |
| Male | -0.050**** | -0.035**** | -0.020**** |
|  | 0.001 | 0.001 | 0.001 |
|  | -0.053,-0.047 | -0.038,-0.032 | -0.022,-0.019 |
| NH White | Ref | Ref | Ref |
| NH Black | -0.071**** | -0.067**** | -0.032**** |
|  | 0.002 | 0.005 | 0.006 |
|  | -0.076,-0.066 | -0.077,-0.056 | -0.044,-0.020 |
| Hispanic | -0.075**** | -0.058**** | -0.047**** |
|  | 0.003 | 0.006 | 0.01 |
|  | -0.081,-0.069 | -0.070,-0.046 | -0.066,-0.028 |
| Asian | -0.029**** | 0.011 | 0.033* |
|  | 0.006 | 0.013 | 0.017 |
|  | -0.042,-0.017 | -0.014,0.037 | -0.001,0.067 |
| Native American | -0.104**** | -0.205**** | -0.218**** |
|  | 0.006 | 0.011 | 0.01 |
|  | -0.117,-0.092 | -0.227,-0.183 | -0.238,-0.198 |
| others | 0.013**** | 0.018**** | 0.014*** |
|  | 0.003 | 0.004 | 0.005 |
|  | 0.006,0.019 | 0.010,0.025 | 0.005,0.023 |
| Northeast | Ref | Ref | Ref |
| Midwest | 0.022**** | -0.004 | -0.011 |
|  | 0.007 | 0.012 | 0.013 |
|  | 0.009,0.035 | -0.028,0.020 | -0.036,0.014 |
| South | -0.002 | -0.005 | 0.022* |
|  | 0.006 | 0.011 | 0.012 |
|  | -0.014,0.011 | -0.026,0.016 | -0.001,0.046 |
| West | -0.024*** | -0.078**** | -0.076**** |
|  | 0.008 | 0.014 | 0.018 |
|  | -0.039,-0.009 | -0.106,-0.050 | -0.111,-0.041 |
| Non-Dual eligible | Ref | Ref | Ref |
| Dual eligible | -0.043**** | -0.070**** | -0.059**** |
|  | 0.003 | 0.005 | 0.007 |
|  | -0.048,-0.038 | -0.080,-0.060 | -0.072,-0.046 |
| Non-ADRD | Ref | Ref | Ref |
| ADRD | -0.010**** | -0.073**** | -0.091**** |
|  | 0.003 | 0.003 | 0.003 |
|  | -0.015,-0.005 | -0.079,-0.067 | -0.097,-0.086 |
| Observations | 1156693 | 965938 | 6676575 |

**Note:** *p < 0.10 **p < 0.05 ***p < 0.01 ****p < 0.001

**Abbreviations:** ame: Average Marginal Effects; se: Standard Error; ci95: 95% Confidence Interval

**Table 9. AWVs Uptake and Medicare Insurance Type: An Ordinary Least Squares (OLS) Linear Regression Model**

|  | Coefficients |
| --- | --- |
|  | b/ci95 |
| Fee-for-Service | Ref |
| Medicare Advantage | 0.042**** |
|  | 0.005 |
|  | 0.032,0.053 |
| 65-74 | Ref |
| 75-84 | 0.005**** |
|  | 0.001 |
|  | 0.003,0.007 |
| 85+ | -0.049**** |
|  | 0.002 |
|  | -0.052,-0.046 |
| Female | Ref |
| Male | -0.026**** |
|  |  |
|  | -0.026,-0.025 |
| NH White | Ref |
| NH Black | -0.026**** |
|  | 0.001 |
|  | -0.027,-0.024 |
| Hispanic | -0.052**** |
|  | 0.008 |
|  | -0.068,-0.037 |
| Asian | 0.019 |
|  | 0.014 |
|  | -0.009,0.048 |
| Native American | -0.185**** |
|  | 0.008 |
|  | -0.201,-0.169 |
| others | 0.014**** |
|  | 0.004 |
| Northeast | 0.006,0.022 |
| Midwest | -0.005 |
|  | 0.012 |
|  | -0.029,0.018 |
| South | 0.017 |
|  | 0.011 |
|  | -0.005,0.039 |
| West | -0.067**** |
|  | 0.016 |
|  | -0.098,-0.036 |
| Non-Dual eligible | Ref |
| Dual eligible | -0.068**** |
|  | 0.006 |
|  | -0.081,-0.055 |
| Non-disease | Ref |
| One disease | 0.170**** |
|  | 0.002 |
|  | 0.166,0.175 |
| Comorbidity | 0.251**** |
|  | 0.003 |
|  | 0.245,0.258 |
| Constant | 0.188**** |
|  | 0.009 |
|  | 0.170,0.206 |
| Observations | 8799206 |

**Note:** *p < 0.10 **p < 0.05 ***p < 0.01 ****p < 0.001

**Abbreviations:** ame: Average Marginal Effects; se: Standard Error; ci95: 95% Confidence Interval

**Table 10. AWVs Uptake and Medicare Insurance Type: Probit Models**

|  | **Model1** | **Model2** | **Model3** | **Model4** |
| --- | --- | --- | --- | --- |
|  | ame/ci95 | ame/ci95 | ame/ci95 | ame/ci95 |
| Fee-for-Service | Ref | Ref | Ref | Ref |
| Medicare Advantage | 0.044**** | 0.048**** | 0.044**** | 0.043**** |
|  | 0.006 | 0.005 | 0.005 | 0.005 |
|  | 0.032,0.055 | 0.038,0.059 | 0.033,0.054 | 0.033,0.053 |
| 65-74 | Ref | Ref | Ref | Ref |
| 75-84 | 0.027**** | 0.027**** | 0.004**** | -0.001* |
|  | 0.001 | 0.001 | 0.001 | 0.001 |
|  | 0.025,0.030 | 0.025,0.029 | 0.003,0.006 | -0.003,0.000 |
| 85+ | -0.026**** | -0.023**** | -0.048**** | -0.045**** |
|  | 0.002 | 0.002 | 0.002 | 0.001 |
|  | -0.029,-0.022 | -0.026,-0.020 | -0.051,-0.045 | -0.048,-0.042 |
| Female | Ref | Ref | Ref | Ref |
| Male | -0.031**** | -0.033**** | -0.027**** | -0.027**** |
|  | 0.001 | 0.001 | 0.001 | 0.001 |
|  | -0.033,-0.030 | -0.035,-0.031 | -0.028,-0.025 | -0.029,-0.026 |
| NH White | Ref | Ref | Ref | Ref |
| NH Black | -0.039**** | -0.043**** | -0.043**** | -0.042**** |
|  | 0.006 | 0.006 | 0.005 | 0.005 |
|  | -0.050,-0.027 | -0.054,-0.032 | -0.054,-0.032 | -0.052,-0.031 |
| Hispanic | -0.086**** | -0.059**** | -0.055**** | -0.054**** |
|  | 0.007 | 0.007 | 0.008 | 0.008 |
|  | -0.101,-0.072 | -0.073,-0.044 | -0.070,-0.040 | -0.070,-0.039 |
| Asian | -0.028** | 0.016 | 0.02 | 0.02 |
|  | 0.014 | 0.015 | 0.015 | 0.015 |
|  | -0.056,-0.001 | -0.014,0.046 | -0.010,0.049 | -0.009,0.050 |
| Native American | -0.225**** | -0.201**** | -0.201**** | -0.202**** |
|  | 0.01 | 0.009 | 0.009 | 0.009 |
|  | -0.245,-0.205 | -0.220,-0.183 | -0.219,-0.183 | -0.220,-0.184 |
| others | 0.002 | 0.011*** | 0.015**** | 0.015**** |
|  | 0.004 | 0.004 | 0.004 | 0.004 |
|  | -0.007,0.011 | 0.004,0.019 | 0.007,0.023 | 0.008,0.023 |
| Northeast | Ref | Ref | Ref | Ref |
| Midwest |  | -0.013 | -0.004 | -0.005 |
|  |  | 0.012 | 0.012 | 0.012 |
|  |  | -0.036,0.011 | -0.028,0.019 | -0.028,0.018 |
| South |  | 0.019* | 0.017 | 0.017 |
|  |  | 0.011 | 0.011 | 0.011 |
|  |  | -0.002,0.041 | -0.004,0.038 | -0.004,0.038 |
| West |  | -0.084**** | -0.067**** | -0.068**** |
|  |  | 0.016 | 0.016 | 0.016 |
|  |  | -0.115,-0.052 | -0.098,-0.037 | -0.099,-0.037 |
| Non-Dual eligible |  | Ref | Ref | Ref |
| Dual eligible |  | -0.055**** | -0.068**** | -0.057**** |
|  |  |  |  | 0.006 |
|  |  | -0.056,-0.054 | -0.069,-0.067 | -0.069,-0.045 |
| Non-disease |  |  | Ref | Ref |
| One disease |  |  | 0.172**** | 0.172**** |
|  |  |  | 0.002 | 0.002 |
|  |  |  | 0.169,0.176 | 0.168,0.176 |
| Comorbidity |  |  | 0.253**** | 0.252**** |
|  |  |  | 0.003 | 0.003 |
|  |  |  | 0.247,0.260 | 0.245,0.258 |
| Non-ADRD |  |  |  |  |
| ADRD |  |  |  | -0.081**** |
|  |  |  |  | 0.002 |
|  |  |  |  | -0.086,-0.076 |
| Observations | 8799206 | 8799206 | 8799206 | 8799206 |

**Note:** *p < 0.10 **p < 0.05 ***p < 0.01 ****p < 0.001

**Abbreviations:** ame: Average Marginal Effects; se: Standard Error; ci95: 95% Confidence Interval
